# Quality of life and mortality in the general population: a systematic review and meta-analysis

**DOI:** 10.1186/s12889-020-09639-9

**Published:** 2020-11-06

**Authors:** Aung Zaw Zaw Phyo, Rosanne Freak-Poli, Heather Craig, Danijela Gasevic, Nigel P. Stocks, David A. Gonzalez-Chica, Joanne Ryan

**Affiliations:** 1 School of Public Health and Preventive Medicine, Monash University, 99 Commercial Road, Melbourne, VIC 3004 Australia; 2 Department of Epidemiology, Erasmus Medical Centre, 3015 GD Rotterdam, The Netherlands; 3 Usher Institute, University of Edinburgh, Teviot Place, Edinburgh, EH8 9AG UK; 4 Discipline of General Practice, Adelaide Medical School, The University of Adelaide, Adelaide, SA 5005 Australia; 5 Adelaide Rural Clinical School, The University of Adelaide, Adelaide, SA 5005 Australia; 6 PSNREC, Univ Montpellier, INSERM, 34000 Montpellier, France

**Keywords:** Quality of life, Life quality, Health-related quality of life, Mortality, Meta-analysis, Predictor, Review

## Abstract

**Background:**

Quality of life (QoL) is multi-dimensional concept of an individual’ general well-being status in relation to their value, environment, cultural and social context in which they live. This study aimed to quantitatively synthesise available evidence on the association between QoL and mortality in the general population.

**Methods:**

An electronic search was conducted using three bibliographic databases, MEDLINE, EMBASE and PsycINFO. Inclusion criteria were studies that assessed QoL using standardized tools and examined mortality risk in a non-patient population. Qualitative data synthesis and meta-analyses using a random-effects model were performed.

**Results:**

Of 4184 articles identified, 47 were eligible for inclusion, involving approximately 1,200,000 participants. Studies were highly heterogeneous in terms of QoL measures, population characteristics and data analysis. In total, 43 studies (91.5%) reported that better QoL was associated with lower mortality risk. The results of four meta-analyses indicated that higher health-related QoL (HRQoL) is associated with lower mortality risk, which was consistent for overall HRQoL (HR 0.633, 95% CI: 0.514 to 0.780), physical function (HR 0.987, 95% CI: 0.982 to 0.992), physical component score (OR 0.950, 95% CI: 0.935 to 0.965), and mental component score (OR 0.980, 95% CI: 0.969 to 0.992).

**Conclusion:**

These findings provide evidence that better QoL/HRQoL was associated with lower mortality risk. The utility of these measures in predicting mortality risk indicates that they should be considered further as potential screening tools in general clinical practice, beyond the traditional objective measures such as body mass index and the results of laboratory tests.

## Background

Quality of life (QoL) is a multi-dimensional concept of an individual’s general well-being status in relation to the value, environment, cultural and social context in which they live [1]. Since QoL measures outcomes beyond biological functioning and morbidity [2], it is recognised as an important measure of overall [1]. The origin of the term QoL dates back to the early 1970s, as a measure of wellness with linkage to health status like diseases or disability [3, 4]. Since then, interest in QoL has increased considerably [5]. As life expectancy increases, more emphasis has been placed on the importance of better QoL, and the maintenance of good health for as long as possible [6–9]. Indeed, global leading health organizations have emphasized the importance of QoL and well-being as a goal across all life stages [10–12].

Moreover, QoL has increasingly been used in the wider context to monitor the efficacy of health services (e.g. patient reported outcome measures, PROMs), to assess intervention outcomes, and as an indicator of unmet needs [13–15]. Several studies have reported that QoL is negatively associated with rehospitalization and death in patients with diseases such as coronary disease [16, 17], and pulmonary diseases [18]. Further, QoL is also predictive of overall survival in patients affected by cancer, chronic kidney disease or after coronary bypass graft surgery [19–22]. In recent years, an increasing number of studies have investigated whether QoL is also a predictor of mortality risk in the general population [23–27].

To date, there has been only one pooled analysis of eight heterogeneous-Finnish cohorts. That study of 3153 older adults, focused exclusively on the prognostic value of the validated 15-dimentional (15D) health-related QoL (HRQoL) measures [28] for predicting all-cause mortality [29]. However, there has been no systematic review investigating the association between QoL measured by different instruments and all-cause mortality in population-based samples which could be used to monitor health changes in the general population. A broad and comprehensive systematic review of the prognostic value of QoL for all-cause mortality prediction is needed to determine the utility of this QoL measure as a potential screening tool in general clinical practice. Therefore, this systematic review and meta-analysis was conducted with the aim of determining whether QoL is predictive of mortality in the general population which includes individuals with or without a range of health conditions.

## Methods

### Search methods

This systematic review and meta-analysis were conducted in accordance with the Preferred Reporting Items for Systematic Reviews and Meta-Analyses (PRISMA) statement [30]. The protocol for this review was registered with the International Prospective Register of Ongoing Systematic Reviews (PROSPERO) [31], under the registration number: CRD42019139994 [32]. The electronic bibliographic databases, MEDLINE, EMBASE and PsycINFO (through OVID) were searched from database inception until June 21, 2019. The search strategy was developed in consultation with a Senior Medical Librarian. The MeSH terms and key-words were developed for MEDLINE (through OVID) and were translated to EMBASE and PsycINFO using the OVID platform (See Supplementary Tables S1-S3, Additional File 1). When the full text of an article was not available, all attempts were made to obtain it by contacting the authors directly. To identify further potentially relevant studies, another search was also developed with those specific QoL / HRQoL measures which were found in this review (See Supplementary Table S4, Additional File 1). Additionally, the bibliography lists of the included articles were also hand searched.

### Inclusion and exclusion criteria

Articles were included if they: (a) involved adults aged 18 years and older; (b) were general population-based samples with or without a range of health conditions; (c) assessed mortality from any cause or cause-specific mortality using a longitudinal design; and (d) included a QoL / HRQoL measure using a standard tool. QoL, the general well-being of individuals, consists of a range of contexts – health, education, employment, wealth, politics and the environment [33]. HRQoL, the self-perceived health status, includes physical, mental, emotional, and social domains [33]. We excluded papers not written in English, reviews, or studies including only specific groups of patients (e.g. patients on dialysis, those with fractures, after surgery, or individuals with a terminal illness).

### Study selection

The screening of articles for eligibility according to title and abstract was undertaken independently by two reviewers (AZZP and HC). All relevant full-text articles were independently reviewed by two reviewers (AZZP and HC) for eligibility against inclusion criteria. The inter-coder reliability among two reviewers (AZZP and HC) was 98%. Discrepancies and disagreements between two reviewers (AZZP and HC) were resolved through discussion with a third reviewer (JR). The screening process was undertaken using Covidence online software [34] and EndNote X9 software.

### Data extraction

A standard data extraction form was used which included the following fields – title, authors, year of publication, setting/country, name of the study and design, sample size, follow-up period, participant characteristics (age and sex), specific QoL measure, cause of death (if available), and results (risk estimates including 95% confidence intervals, CI) which were standardized in term of 1-unit increase or 1-SD increase for continuous risk estimate, or high vs. low for categorical risk estimates. The first reviewer (AZZP) completed the data extraction form and a second reviewer (HC) verified the extracted information. All efforts were made to contact authors when there was missing information.

### Quality appraisal

The quality of included studies was appraised using ‘the Newcastle – Ottawa Quality Assessment Scale (NOS)’ [35]. The NOS includes eight items, categorized into three dimensions (a) Selection, (b) Comparability, and (c) Outcome. The NOS scale uses a star system to evaluate the quality of each study, and they can be accredited a maximum of one star for each item within the Selection and Outcome dimension and two stars for the Comparability item. When considering the comparability of each study, a star was provided for studies which controlled for relevant covariates – age, sex (where appropriate), socioeconomic status or proxy (including socioeconomic position, education level or income), and some measure of co-morbidity (for example a specific health condition). An additional star was given for studies which considered other factors associated with QoL and mortality, including clinical measures, BMI, or lifestyle factors (i.e. smoking, alcohol, physical activity). The range of NOS scoring was from 0 to 9 stars, with higher scores indicating less susceptibility to bias. The methodological quality of included studies was rated by one reviewer (AZZP) and verified by a second reviewer (HC). Disagreements were resolved through discussion with a third reviewer (JR).

### Data synthesis

The clinical and methodical heterogeneity of the studies was examined, in particular considering the measure of QoL used, and the effect estimates reported (Hazard Ratio (HR), Relative Risk (RR) or Odds Ratio (OR)). Where studies were considered too methodically heterogeneous to enable pooling, the results were summarized quantitatively in tables according to related categories with risk estimates; and 95% CIs.

### Meta-analysis

A meta-analysis was performed when there was a sufficient number of studies (four or more) which used the same domain of QoL measure and equivalent effect estimate parameters. In the present study, four meta-analyses were conducted for a pooled risk estimate of studies using (a) physical component score (PCS) of 36-item Short Form (SF-36) and OR / RR; (b) physical function domain of SF-36 and HR; (c) mental component score (MCS) of SF-36 and OR / RR; and (d) the 15-dimensional measure (15D) and HR. A DerSimonian-Laird random-effects model was chosen given heterogeneity in the studies in terms of population characteristics and varying health status. When more than one risk estimate was reported in the study, the fully adjusted/final regression model was included. In addition, when the included studies from the same cohorts with the same follow-up were eligible for meta-analysis, only one study with larger sample size was chosen for meta-analysis. Effect estimates were standardized where possible, so all values corresponded to a 1-unit increase in SF-36 or a 1-SD increase in 15D (single index number). A pooled risk estimates of less than one indicates a decreased risk of mortality with higher QoL. Statistical heterogeneity was evaluated by using the I^2^ statistic, and the results were interpreted based on the Cochrane guidelines (0–40% = no heterogeneity; 30–60% = moderate heterogeneity; 50–90% = substantial heterogeneity; and 75–100% = considerable heterogeneity) [36]. In addition, when the I^2^ statistic showed considerable heterogeneity (≥ 75%), the influence of individual studies on the pooled risk estimate was assessed using the metaninf command of STATA. Funnel plots and Egger’s test were used to assess publication bias. Data analysis was undertaken using STATA statistical software, version 15.0 (StataCorpLP, College Station, TX, USA).

## Results

### Search result

**Fig. 1:**
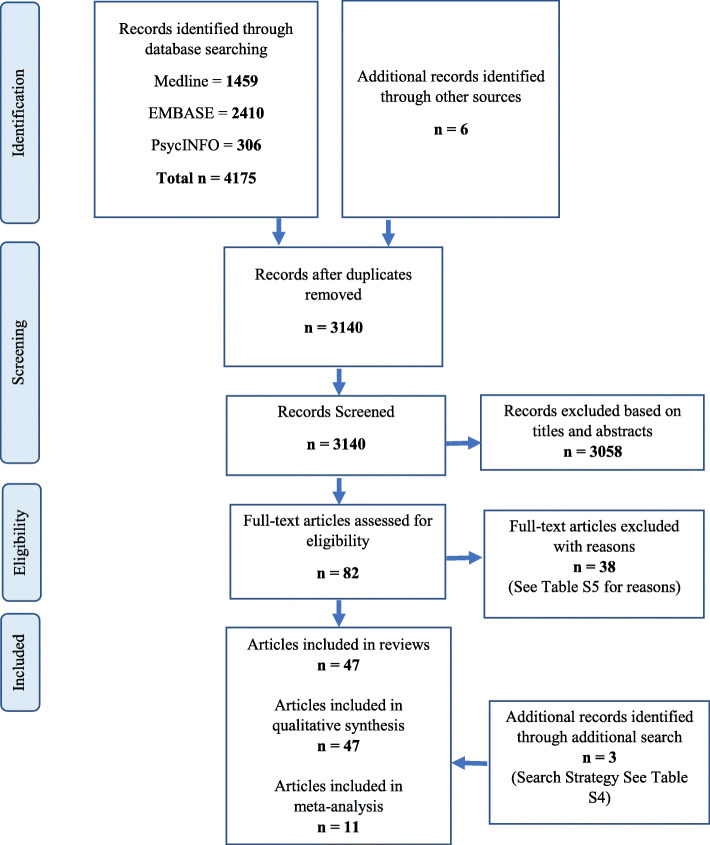
Flow Diagram of Review Process

A total of 4175 articles were identified from the systematic database search, and six additional articles were found via searching the reference list of included articles (Fig. 1). After removing duplicates, 3140 records remained for review. After title and abstract screening, 3058 articles were excluded and the full-text of the remaining 82 articles were evaluated for eligibility. A total of forty-four (44) articles met all inclusion criteria. Excluded articles with reasons for exclusion are presented in Supplementary Table S5, Additional File 1. Moreover, three articles from additional search were also added in this review. Therefore, a total of forty-seven (47) articles were included in this systematic review.

### Description of included studies

Table 1 presents the characteristics of the 47 included studies. The earliest study was published in 1993 while the remaining included articles were published between 2002 and 2019, with 28% published in the past 5 years. All studies except the retrospective cohort study of Ul-Haq et al., [75] were prospective cohort studies. The included studies were conducted in USA (34%), UK (9%), Australia (6%), Canada (6%), Spain (6%), Taiwan (6%), Belgium (4%), Finland (4%), Scotland (4%), Sweden (4%), Bangladesh (2%), China (2%), Germany (2%), South Korea (2%), Italy (2%), Norway (2%), and South Africa (2%). The sample sizes of the included studies ranged from 171 [41] to 559,985 [40]; 14 studies had a sample size of less than 1000, 17 studies between 1000 and 10,000, 13 studies between 10,000 and 100,000, and the remaining three studies [38, 40, 53] has a sample size of more than 100,000 participants. Five studies included only males [41, 42, 54, 71, 73] and three studies only females [56, 59, 74]. The remaining 39 studies recruited between 3 to 78% of women. The follow-up periods of the studies varied between 9 months [72] and 18 years [73].

**Table 1:** Characteristics of the 47 included studies

Authors and Year	Setting - Country	Study Name and Design	Sample Size	Follow-up in years	Participants (Age in Range or Mean (SD), Female %)	QoL Measure	Type of Death	Comparison	Risk estimate (95% CI)	Adjustment
Bjorkman et al. 2019 [37]	Finland	Porvoo Sarcopenia and Nutrition Trial, Prospective	428	4 yrs	75 yrs. and + 66.59%	RAND-36 PF	all-cause	HR, 1-unit increase	PF: 0.988 (0.979–0.997)	age, sex, comorbidity and CRi-SMI
Brown et al. 2015 [38]^a^	USA	Medicare Health Outcomes Survey (Cohort 6–8), Prospective	191,001	2.5 yrs	65 yrs. and + 58.30%	CDC HRQOL-4	all-cause	HR, Excellent vs. Poor HR, 0 days vs. 21–30 days	GH: 0.24 (0.21–0.27) Days of not good in Physical Health 0.82 (0.77–0.88) Days of not good in Mental Health 1.12 (1.04–1.22) Days of activity limitation 0.74 (0.68–0.79)	age, sex, race/ethnicity, education, income, range of other health and lifestyle factors
Cavrini et al. 2012 [39]	Italy	Pianoro Study, Prospective	5256	2 yrs	65 yrs. and + 55.3%	EQ-5D	all-cause	HR, 1-unit increase	0.42 (0.35–0.50)	sex, age, BMI, education, health and lifestyle factors
Chwastiak et al. 2010 [40]	USA	1999 Large Health Survey of Veteran Enrollees, Prospective	559,985	9 yrs	64.1 (12.9) yrs4.1%	SF-36 PCS	all-cause	HR, 1-unit increase	PCS: 0.97 (0.96–0.98)	age, race, sex, education, disability, comorbidity, BMI, lifestyle factors
De Buyser et al. 2016 [41]^a^	Belgium	Prospective cohort	171	15 yrs	71 yrs. and + 0%	SF-36 PFI	all-cause	HR, 1-unit increase	PF: 1.01 (0.99–1.02)	age, polypharmacy, depression, and disability
De Buyser et al. 2013 [42]^a^	Belgium	Prospective cohort	352	15 yrs	71 to 86 yrs0%	SF-36 PFI	all-cause	HR, 1-unit increase	PF: 0.992 (0.986–0.999)	age, BMI and smoking
DeSalvo et al. 2005 [43]	USA	VAAC Quality Improvement Project, Prospective	21,732	1 yr	64 (12) yrs3.6%	SF-36 PCS and MCS	all-cause	AUC	PCS: 0.73 (0.71–0.75) MCS: 0.68 (0.66–0.70)	age
Dominick et al. 2002 [44]^a^	USA	Pennsylvania’s Pharmaceutical Assistance Contract for the Elderly, Prospective	84,065	1 yr	78.7 (6.9) yrs. 78.0%	Core CDC HRQOL items	all-cause	RR, Excellent vs. Poor RR, 0 days vs. 21–30 days	GH: 0.24 (0.17–0.33) Days of not good in Physical Health 0.42 (0.38–0.45) Days of not good in Mental Health 0.53 (0.50–0.59) Days of activity limitation 0.40 (0.37–0.42)	age, sex, race, marital and residential status, income and comorbidity
Dorr et al. 2006 [45]^a^	USA	Intermountain Health Care Network, Prospective	2166	2.3 yrs	77.9 (6.8) yrs54.9%	SF-12 PCS and MCS	all-cause	OR, Quartile 4 (Highest) vs. Quartile 1 (Lowest)	PCS: 0.16 MCS: 0.40	age, sex, and comorbidity
Drageset et al. 2013 [46]	Norway	Study of Nursing Home Residents without cognitive impairment (2004–2005), Prospective	227	5 yrs	65 to 95 yrs. and + 72.25%	SF-36 PCS and MCS	all-cause	HR, 1-unit increase	PF: 0.99 (0.98–0.99)	age, sex, marital status, education and comorbidity
Fan et al. 2004 [24]^a^	USA	VAAC Quality Improvement Project, Prospective	7702	1 yr	65.4 (10.6) yrs. 3.4%	SF-36 PCS and MCS	all-cause	OR, 1-unit increase	PCS: 0.956 (0.943–0.969) MCS: 0.981 (0.971–0.990)	age, site, distance to the VA, and comorbidity
Fan et al. 2006 [47]	USA	VAAC Quality Improvement Project, Prospective	14,192	3 yrs	64.4 (11.3) yrs. 3.5%	SF-36 PCS and MCs	all-cause	AUC	PCS: 0.721 (0.708–0.733) MCS: 0.689 (0.675–0.702)	age and sex
Feeny et al. 2012 [48]	Canada	1994/95 Canadian National Population Health Longitudinal Survey, Prospective	12,375	12 yrs	18–80 yrs. + 52%	HUI3	all-cause	HR, 1-level increase	Hearing: 0.18 (0.06–0.57) Ambulation: 0.10 (0.04–0.23) Pain: 0.53 (0.29–0.96)	age, sex, socioeconomic, disease condition, and lifestyle factors
Forsyth et al. 2018 [27]^a^	Australia	RCT of a case Management Intervention for Adult transitioning from prison to the community, Prospective	1320	4.7 yrs	32.7 (11.1) yrs. 21.10%	SF-36 PCS and MCS	all-cause	HR, High vs. Low	PCS: 0.48 (0.18–1.20) MCS: 0.38 (0.16–0.91) ^a^(CI is 99%CI)	age, sex and indigenous status
Franks et al. 2003 [49]^a^	USA	Household Survey component of the National Medical Expenditure, Prospective	21,363	5 yrs	21 yrs. + 55.39%	SF-20	all-cause	HR, 1-point increase	HP: 0.993 (0.990–0.996) PF: 0.995 (0.992–0.997) RF: 0.996 (0.994–0.998) MH: 1.00 (0.996–1.003)	age, sex, race, ethnicity, education and income
Gomez-Olive et al. 2014 [25]^a^	South Africa	Population under the Agincourt Health and Demographic Surveillance System, Prospective	4047	3 yrs	50 yrs. + 75.8%	WHO QOL	all-cause	HR, Highest vs. Lowest	Overall: 0.61	age, sex, education and union status, HH assets, and Disability Assessment
Han et al. 2009 [50]	South Korea	Korea Longitudinal Study on Health and Aging, Prospective	944	3.25 yrs. (median)	76.0 (8.6) yrs. 54.9%	SF-36 PCS and MCS (K.V)	all-cause	HR, Tertile 3 (High) vs. Tertile 1 (Low)	PCS: 0.35 (0.19–0.64) MCS: 0.39 (0.22–0.70)	age, sex, smoking, range of serum measures
Haring et al. 2011 [51]^a^	Germany	Population-based Study of Health in Pomerania, Prospective	4261	9.7 yrs. (mean)	20–79 yrs. 50.93%	SF-12 PCS and MCS	all-cause	HR, Highest Quartile vs. Lowest Quartile	PCS: 0.56 (0.42–0.75)^##^ PCS: 0.63 (0.47–0.84)# MCS: 0.94 (0.73–1.22)^##^ MCS: 1.04 (0.81–1.35)#	age, sex, ## behavioural factors, # comorbidities
Higueras-Fresnillo et al. 2018 [52]^a^	Spain	UAM Cohort, Prospective	3922	14 yrs. (median)	71.82 (7.94) yrs. 56.38%	SF-36 PCS and MCS	all-cause	HR, Good vs. Poor	Physical: 0.74 (0.65–0.85) Mental: 0.85 (0.74–0.98) Social: 0.73 (0.63–0.85)	age, sex, education, lifestyle factors, BMI, waist circumference, comorbidity
Jia et al. 2018 [53]^a^	USA	Medicare Health Outcomes Survey Cohort 15, Prospective	105,473	2 yrs	65 yrs. + 58.30%	SF-6D and dEQ-5D	all-cause	HR, 1st Quintile vs. 5th Quintile	SF-6D: 0.77 (0.71–0.80) dEQ-5D: 0.45 (0.43–0.49)	age, sex, socioeconomic, marital status, smoking, BMI, chronic conditions
Kao et al. 2005 [54]	Taiwan	Prospective Cohort	689	2 yrs	65 yrs. + 0%	WHOQOL-(BREF)	all-cause	RR, 1-point change	Overall: 0.99 (0.77–1.26)	unadjusted RR
Kaplan et al. 2007 [55]	Canada	1994/95 Canadian National Population Health Longitudinal Survey, Prospective	12,375	8 yrs	18–80 yrs. + 52%	HUI3	all-cause	HR, 1-unit increase	0.61 (0.42–0.89)	age, sex, socioeconomics, other social/health, lifestyle factors
Kroenke et al. 2008 [56]	USA	Nurses’ Health Study, Prospective	40,337	2.8 to 12 yrs	46–71 yrs. 100%	SF-36 PCS and MCS	all-cause	RR^###^, Severe Decline vs. No Change RR^####^, Improve vs. No Change	Change in PCS 3.32^###^ (2.45–4.50) 0.72^####^ (0.56–0.91) Change in MCS 1.86^###^ (1.17–2.97) 0.77^####^ (0.63–0.95)	age, baseline HRQoL, menopausal status, social integration, BMI, educational, husbands’ education, lifestyle factors, PCS/MCS
Lawler et al. 2013 [57]	USA	Oklahoma Longitudinal Assessment of Health Outcomes of Mature Adults Studies, Prospective	852	5 yrs	65 yrs. + 56.81%	SF-36 PCS and MCS	all-cause	HR, 1-unit increase	PF: 0.98 (0.97–0.98) Bodily Pain: 1.01 (1.00–1.01)	age, sex, socioeconomic, BMI, morbidity, functional status, having a confidant
Lee et al. 2012 [58]^a^	Taiwan	Elderly Nutrition and Health Survey, Prospective	1435	7.9 yrs	65–97 yrs. 48.50%	SF-36 PCS (T.V 1.0)	all-cause	HR, Highest PF vs. Lowest PF	PF: 0.29 (0.19–0.45)	age
Leigh et al. 2015 [59]	Australia	Australian Longitudinal Study on Women’s Health, Prospective	10,721	15 yrs	70–75 yrs. 100%	SF-36 Vitality, Mental and PF	all-cause	HR, 1-unit increase	PF: 0.992 (0.990–0.994) Mental:1.0 (0.997–1.002) Vitality: 1.0 (0.998–1.002)	age, socioeconomic, BMI, sleep, disease count, and other health factors
Liira et al. 2018 [29]	Finland	a. The Helsinki Businessmen Study (HBS) b. Spousal caregivers of people with dementia c. Nursing home residents d. Older persons suffering from loneliness e. Population Sample	a = 733 b = 209 c = 326 d = 208 e = 901	2 yrs	a. 77 (4) yrs. 0% b. 75 (7) yrs. 64.6% c. 84 (7) yrs. 69.9% d. 80 (4) yrs. 75% e. 85 (5) yrs. 75.1%	The 15D	all-cause	HR, 1SD (0.14) increase	a. 0.43 (0.31–0.63) b. 1.06 (0.43–2.63) c. 0.69 (0.58–0.85) d. 0.94 (0.47–1.87) e. 0.62 (0.49–0.72)	age and sex
Masel et al. 2010 [60]	USA	Hispanic Established Population for Epidemiologic Study of the Elderly, Prospective	1008	2 yrs	74–101 yrs. 63.2%	SF-36 PCS and MCS	all-cause	OR, 1-point increase	PCS: 0.962 (0.941–0.984) MCS: 0.996 (0.974–1.018)	age, sex, education, marital status, financial strain, chronic illness, smoking, BMI, and frailty
Mold et al. 2008 [61]	USA	Oklahoma Longitudinal Assessment of Health Outcomes of Mature Adults Studies, Prospective	604	5 yrs	65 yrs. + 56%	SF-36 PF and bodily pain	all-cause	HR, 1-unit increase	PF: 0.98 (0.97–0.99)	education, income, smoking, initial and instrumental activity of daily living, health utilities / conditions
Munoz et al. 2011 [62]	Spain	Prospective Cohort	3724	6.3 yrs. (median)	35–74 yrs. 51.9%	SF-12 PCS and MCS	all-cause	HR, 3rd Tertile vs.1st Tertile (Low)	PCS: 0.58 (0.39–0.87) MCS: 0.99 (0.69–1.42)	age, sex, marital status, education and cardiovascular risk factors
Murray et al. 2011 [63]	Scotland	Lothian Birth Cohort 1921, Prospective	448	9 yrs	79 yrs. 56.70%	26-item WHOQOL-BREF	all-cause	HR, 1 tertile increase / 1-point increase	Overall: 0.84 (0.67–1.05) GH: 0.75 (0.64–0.89) Physical: 0.90 (0.86–0.95) Psychological: 0.98 (0.91–1.06) Social: 0.97 (0.91–1.04) Environment: 0.96 (0.89–1.03)	age and sex
Myint et al. 2006 [64]^a^	UK	European Prospective Investigation into Cancer -Norfolk, Prospective	17,777	6.5 yrs. (mean)	41–80 yrs. 56.25%	SF-36 PCS (UK.V)	all-cause	RR, Quintiles 5 (Highest) vs. Quintiles 1	PCS Men: 0.47 (0.33–0.65) Women: 0.41 (0.27–0.64)	age, BMI, SBP, blood cholesterol, smoking, diabetes and social class
Myint et al. 2007 [65]^a^	UK	European Prospective Investigation into Cancer -Norfolk, Prospective	17,777	6.5 yrs. (mean)	40–79 yrs. 56.25%	SF-36 MCS (UK.V)	all-cause	HR, 1-point increase	MCS: 0.987 (0.981–0.993)	age, sex, PCS, lifestyle, BMI, SBP, blood cholesterol, diabetes, and social class
Myint et al. 2010 [26]^a^	UK	European Prospective Investigation into Cancer -Norfolk, Prospective	17,736	6.5 yrs. (mean)	40–79 yrs. 56.23%	SF-6D (UK.V)	all-cause	HR, 1 SD (0.12-point) increase	0.74 (0.69–0.79)	age, sex, BMI, SBP, blood cholesterol, diabetes, smoking, and social class
Nilsson et al. 2011 [66]^a^	Sweden	Inhabitants in the Swedish city of Vasteras, Prospective	417	10 yrs	75 yrs. 51.08%	PGWB	all-cause	RR, 1-unit change	Global Score Men: 0.984 (0.969–0.998) Women: 0.994 (0.978–1.010)	for men: smoking, obesity, living alone and other health conditions
Otero-Rodriguez et al. 2010 [67]^a^	Spain	Spanish Population-Based Cohort, Prospective	2373	6 yrs	60 yrs. + 57.5%	SF-36 PCS and MCS	all-cause	HR, 1-point increase	PCS: 0.952 (0.935–0.969) MCS: 0.990 (0.976–1.006)	sex, age, HRQOL, education, marital status, BMI, other health and lifestyle factors, PCS/MCS
Perera et al. 2005 [68]^a^	USA	Prospective cohort	439	5 yrs	65 yrs. + 44.40%	SF-36 PF	all-cause	HR, 1-point increase	PF: 0.991 (0.945–1.036)	age, sex, measure of change, number of comorbid domains, hospitalization
Razzaque et al. 2014 [69]^a^	Bangladesh	Matlab HDSS, Prospective	4037	2 yrs	50 yrs. + 50.06%	WHOQOL	all-cause	RR, Good/Very Good vs. Bad/Very Bad	Men: 0.26 (0.16–0.41) Women: 0.30 (0.10–0.86)	age and socio-demographic variables
Singh et al. 2005 [70]^a^	USA	Prospective	40,508	1 yr	64.5 (13.7) yrs. 4.2%	SF-36 PCS and MCS (V.V)	all-cause	OR, 1-point increase	PCS: 0.933 (0.926–0.941) MCS: 0.968 (0.962–0.973)	age, sex, socioeconomic, smoking, VA eligibility status, and prior healthcare utilization
St.John et al. 2018 [71]^a^	Canada	Manitoba Follow-up Study, Prospective	734	9 yrs	85.5 (3.0) yrs. 0%	SF-36 PCS and MCS	all-cause	RR, High vs. Low	PCS: 0.50 (0.38–0.64) MCS: 0.55 (0.40–0.76)	age
Sutcliffe et al. 2007 [72]	UK	Prospective	308	0.75 yrs	60–90 yrs. + 68.8%	LQOLP-R - Spitzer	all-cause	HR, increased score	0.9805 (0.9704–0.9907)	unadjusted
Tibblin et al. 1993 [73]	Sweden	Study of men born in 1913, Prospective	787	18 yrs	50 yrs. + 0%	Goteborg QoL	all-cause	No Data	Only Health variable was significantly related to mortality	health, physical fitness, and appetite
Tice et al. 2006 [74]	USA	B-FIT, Prospective	17,748	9 yrs	55–80 yrs. + 100%	SF-20 PF	all-cause	HR, Highest vs. Lowest	PF: 0.70 (0.60–0.90)	age, other health and lifestyle factors
Tsai et al. 2007 [23]^a^	Taiwan	A 2000 Population-based survey in Taiwan, Prospective	4424	3 yrs	65 yrs. +	SF-36 PCS and MCS	all-cause	RR, 1-point increase	PCS: 0.954 (0.941–0.968) MCS: 0.985 (0.971–0.999)	age, sex, feel tired, other health and lifestyle factors
Ul-Haq et al. 2014 [75]^a^	Scotland	Scottish Health Survey 2003, Retrospective	5272	7.6 yrs. (mean)	20–65 yrs. + 54.80%	SF-12 PCS and MCS	all-cause	HR, Best vs. Worst	PCS: 0.36 (0.22–0.57) MCS:0.80 (0.61–1.05)	age, sex, SIMd, education, BMI, other health and lifestyle factors
Williams et al. 2012 [76]^a^	Australia	Australia Diabetes, Obesity and Lifestyle study, Prospective	9979	7.4 yrs	25 yrs. + 55.00%	SF-36 PCS and MCS	all-cause	HR, 1-point change	PF: 0.983 (0.979–0.987) RP: 0.995 (0.993–0.997) Bodily Pain: 0.996 (0.992–0.999) GH: 0.985 (0.980–0.990) Vitality: 0.992 (0.987–0.996) Social F: 0.993 (0.990–0.996) RE: 0.999 (0.996–1.001) MH: 0.999 (0.994–1.004)	age, sex, BMI, smoking, heath conditions, serum measures
Xie et al. 2014 [77]^a^	China	PRC-USA Study, Prospective	1739	10.1 yrs. (median)	57.7 (8.4) yrs. 64.2%	Chinese (QOL-35)	all-cause	HR, Upper 50% vs. Lower 50%	0.69 (0.49–1.00)	age, sex, social-economic, other health and lifestyle factors

*AUC* Area under curve; *BMI* Body Mass Index; *CDC HRQOL-4* Core CDC Healthy Days Measures HRQOL-4; *Chinese (QOL-35)* Chinese 35-item Quality of Life Instrument; *CRi-SMI* Calf Intracellular Resistance Skeletal Muscle Index; *EQ-5D* the EuroQoL-5 Dimension; *GH* General Health; *HUI3* The Health Utilities Index Mark 3 Version; *HH* Household; *HP* Health Perceptions; *HR* Hazard Ratio; *K. V* Korea Version; *LQOLP-R* – *Spitzer* Lancashire Quality-of-Life Profile-Residential incorporated the Spitzer Uniscale; *MCS* Mental Component Score; *MH* Mental Health; *OR* Odds Ratio; *PCS* Physical Component Score; *PF* Physical Functioning; *PGWB* Psychological General Well-Being; *QoL* Quality of Life; *RE* Role-Emotional; *RF* Role Function; *RP* Role Physical; *RR* Relative Risk; *SF-36* Short Form 36; *SF-20* Short Form 20; *SF-12* Short Form 12; *SF-6D* Short-Form Six Dimension Utility Index; *SBP* Systolic Blood Pressure; *Social F* Social Functioning; *SIMd* Scottish Index of Multiple deprivation; *The 15D* The 15 dimensional instrument; *T. V* Taiwan Version; *UK* United Kingdom; *UK. V* UK Version; *USA* United States of America; *VA* Veterans Affairs; *V. V* Veterans Version;

Study Abbreviation; *B-FIT* Breast and Bone Follow-up Study of the Fracture Intervention Trial; *Matlab HDSS* Matlab Health and Demographic Surveillance System of the International Centre for Diarrhoeal Disease Research; *PRC-USA Study* People’s Republic of China-United States of America Chinese Collaborative Study of Cardiovascular and Cardiopulmonary Epidemiology; *VAAC* Veterans Affairs Ambulatory Care;

^a^where studies report reverse association or risk estimate per more than 1-unit increase, the risk estimates were standardised per 1-unit increase or 1-SD increase or high vs. low for the purpose of consistency across the table

This review included a variety of different QoL measures and half of the included studies (24 studies) measured QoL using the Short Form 36 (SF-36) (Tables 1 and 2). Of the 47 articles included in this review (Table 1), some studies involved the same cohorts and, in several cases, likely the same participants. Subsequent publications often reported effect estimates over different lengths of follow-up or using different QoL tools. Two published articles of De Buyser et al. reported the results of the same population-based cohort study [41, 42], three published articles by De Salvo et al. and Fan et al. were from the same study and included participants enrolled in the Veterans Affairs Ambulatory Care Quality Improvement Project [24, 43, 47], two published studies of Mold et al. and Lawler et al. used the same community-dwelling cohort [57, 61], two published studies of Higueras-Fresnillo et al. and Otero-Rodriguez et al. were from the same Spanish cohort [52, 67], two published studies of Feeny et al. and Kaplan et al. were from the same Canadian cohort [48, 55]; and Myint et al. published three articles [26, 64, 65] with different perspectives on the same population-based study. Additionally, Liira et al.’s study [29], included eight individual cohorts, however, only five of the cohorts met the inclusion criteria for this current systematic review, and thus are shown in Table 1.

**Table 2:** Quality of life scale included in the systematic review

QoL Scale		Study
Short Form Health Survey scales	SF-36, SF-20, SF-12, RAND-36	Study [23, 24, 27, 37, 40–43, 45–47, 49–52, 56–62, 64, 65, 67, 68, 70, 71, 74–76]
World Health Organization questionnaires	WHOQOL, WHOQOL-BREF	Study [25, 54, 63, 69]
Centre for Diseases Control and Prevention Health Related Quality of Life scale	CDC HRQOL	Study [38, 44]
Six Dimensions Short Form Scale	SF-6D	Study [26, 53]
Euro Quality of Life scale	EQ-5D	Study [39, 53]
Health Utilities Index 3	HUI3	Study [48, 55]
Psychological General Well-Being Index	PGWB	Study [66]
15-dimensional index	15D	Study [29]
Goteborg Quality of Life Instrument	Goteborg QoL	Study [73]
Lancashire Quality of Life Profile-Residential incorporated the Spitzer Uniscale	LQOLP-Residential incorporated the Spitzer Uniscale	Study [72]
Chinese 35-Item Quality of Life Instrument	Chinese QOL-35	Study [77]

### Risk of Bias assessment

The methodological quality of included studies based on NOS ranged between five and nine stars. Among the included studies, seven were of high methodological quality, with nine stars. Across the ten studies with less than seven stars, they were scored most poorly on the items assessing how representative the cohort was in relation to the overall population being sampled and whether they adjusted for potential confounding factors in their analysis (See Supplementary Table S6-S7, Additional File 1).

### Qualitative synthesis

Of the total 47 included studies, 43 (91.5%) studies reported for at least one of the domains examined, that better QOL was associated with lower mortality risk (Table 1). Of 33 studies which assessed physical HRQoL (nine exclusively assessed physical HRQoL), 30 studies (91%) reported better HRQoL was associated with lower mortality risk. Among the 23 studies which examined mental HRQoL (one exclusively assessed MCS), 13 studies (57%) reported that higher mental HRQoL was associated with decreased mortality risk (Table 1). The five studies [49, 52, 57, 59, 76] that measured HRQoL using SF-36 or SF-20 reported not only the physical functioning and mental health domains, but also general health perception, bodily pain, vitality, and social functioning. The findings were generally consistent in general health perception and social functioning; and it was reported that better level of general health perception and social functioning was associated with decreased mortality risk (Table 1).

The mortality risk estimates of the studies which were not included in the meta-analyses are shown in Tables 3, 4 and 5. The 18 out of 20 studies which measured the PCS using the SF-36 or SF-12 or the physical functioning subscale using SF-36, RAND-36, or SF-20 reported these to be a predictor of mortality risk, with better physical health being associated with lower mortality risk (Table 3). Nine out of 16 studies which assessed the MCS or mental health subscale using SF-36 or SF-12, showed that better mental health was associated with lower mortality risk (Table 4). The 12 out of the 15 studies that measured the association between QoL and mortality risk, found that higher QoL scores were associated with lower mortality risk (Table 5).

**Table 3:** Physical component score / physical functioning as predictors of all-cause mortality

Author (Year)	Comparison	Effect estimate (95% CI)
**SF – 36 Physical Component Score (continuous)**		
Chwastiak et al. 2010 [40]	HR, 1-unit increase	0.97 (0.96–0.98)
DeSalvo et al. 2005 [43]	AUC	0.73 (0.71–0.75)
Fan et al. 2006 [47]	AUC	0.721 (0.708–0.733)
Otero-Rodriguez et al. 2010^f^ [67]	HR, 1-unit increase	0.952 (0.935–0.969)
**SF-36 Physical Function Scale (continuous)**		
De Buyser et al. 2016 ^a,^^f^ [41]	HR, 1-unit increase	1.01 (0.99–1.02)
Mold et al. 2008 ^b^ [61]	HR, 1-unit increase	0.98 (0.97–0.99)
**RAND-36 Physical Function Scale (continuous)**		
Bjorkman et al. 2019 [37]	HR, 1-unit increase	0.988 (0.979–0.997)
**SF – 36 Physical Component Score (categorised)**		
Forsyth et al. 2018^f^ [27]	HR, High vs. Low	0.48 (0.18–1.20) ^e^
Han et al. 2009 [50]	HR, Tertile 3 High vs. Tertile 1Low	0.35 (0.19–0.64)
Higueras-Fresnillo et al.2018^f^ [52]	HR, Good vs. Poor	0.74 (0.65–0.85)
Myint et al. 2006^f^ [64]	RR, Quintile 5 Highest vs. Quintile 1 Lowest	0.47 (0.33–0.65) Men 0.41 (0.27–0.64) Women
St. John et al. 2018^f^ [71]	RR, High vs. Low	0.50 (0.38–0.64)
**SF – 36 Physical Functioning (categorised)**		
Lee et al. 2012^f^ [58]	HR, Highest vs. Lowest	0.29 (0.19–0.45)
**SF – 36 Change in Physical Component Score (categorised)**		
Kroenke et al. 2008 [56]	RR, Severe Decline vs. No Change	3.32 (2.45–4.50)
	RR, Improvement vs. No Change	0.72 (0.56–0.91)
**SF – 20 Physical Function Scale (continuous)**		
Franks et al. 2003^f^ [49]	HR, 1-point increase0.995 (0.992–0.997)	0.995 (0.992–0.997)
**SF – 20 Physical Function Scale (categorised)**		
Tice et al. 2006 [74]	HR, Highest vs. Lowest	0.70 (0.60–0.90)
**SF – 12 Physical Component Score (categorised)**		
Dorr et al. 2006^f^ [45]	OR, Highest Quartile vs. Lowest Quartile	0.16
Haring et al. 2011^f^ [51]	HR, Highest Quartile vs. Lowest Quartile	0.56 (0.42–0.75) ^c^ 0.63 (0.47–0.84) ^d^
Munoz et al. 2011 [62]	HR, 3rd Tertile vs. 1st Tertile	0.58 (0.39–0.87)
UI-Haq et al. 2014^f^ [75]	HR, Best Quintile vs. Worst Quintile	0.36 (0.22–0.57)

^a^De Buyser et al. (2016) and De Buyser et al. (2013) were from the same study. De Buyser et al. (2013) was included in meta-analysis

^b^Lawler et al. (2013) and Mold et al. (2008) were from the same study. Lawler et al. (2013) was included in meta-analysis

^c^behavioural factors adjusted

^d^comorbidities adjusted

^e^ CI is 99% CI

^f^where studies report reverse association or risk estimate per more than 1-unit increase, the risk estimates were standardised per 1-unit increase or 1-SD increase or high vs. low for the purpose of consistency across the table

*AUC* Area under curve

**Table 4:** Mental component score / mental health as predictors of all-cause mortality

Author (Year)	Comparison	Effect estimate (95% CI)
**SF – 36 Mental Component Score (continuous)**		
DeSalvo et al. 2005 [43]	AUC	0.68 (0.66–0.70)
Fan et al. 2006 [47]	AUC	0.689 (0.675–0.702)
Myint et al. 2007^d^ [65]	HR, 1-unit increase	0.987 (0.981–0.993)
Otero-Rodriguez et al. 2010^d^ [67]	HR, 1-unit increase	0.990 (0.976–1.006)
**SF – 36 Mental Health (continuous)**		
Leigh et al. 2015 [59]	HR, 1-unit increase	1.00 (0.997–1.002)
Williams et al. 2012^d^ [76]	HR, 1-point-change	0.999 (0.994–1.004)
**SF – 36 Mental Component Score (categorised)**		
Forsyth et al. 2018^d^ [27]	HR, High vs. Low	0.38 (0.16–0.91) ^c^
Han et al. 2009 [50]	HR, Tertile 3 High vs. Tertile 1Low	0.39 (0.22–0.70)
Higueras-Fresnillo et al. 2018^d^ [52]	HR, Good vs. Poor	0.85 (0.74–0.98)
St. John et al. 2018^d^ [71]	RR, High vs. Low	0.55 (0.40–0.76)
**SF – 36 Change in Mental Component Score (categorised)**		
Kroenke et al. 2008 [56]	RR, Severe Decline vs. No Change RR, Improvement vs. No Change	1.86 (1.17–2.97) 0.77 (0.63–0.95)
**SF – 20 Physical Function Scale (continuous)**		
Franks et al. 2003^d^ [49]	HR, 1-point increase	1.00 (0.996–1.003)
**SF – 12 Mental Component Score (categorised)**		
Dorr et al. 2006^d^ [45]	OR, Highest Quartile vs. Lowest Quartile	0.40
Haring et al. 2011^d^ [51]	HR, Highest Quartile vs. Lowest Quartile	0.94 (0.73–1.22) ^a^
		1.04 (0.81–1.35) ^b^
Munoz et al. 2011 [62]	HR, 3rd Teritle vs. 1st Tertile	0.99 (0.69–1.42)
UI-Haq et al. 2014^d^ [75]	HR, Best Quintile vs. Worst Quintile	0.80 (0.61–1.05)

^a^behavioural factors adjusted

^b^comorbidities adjusted

^c^ 99% CI

^d^where studies report reverse association or risk estimate per more than 1-unit increase, the risk estimates were standardised per 1-unit increase or 1-SD increase or high vs. low for the purpose of consistency across the table

*AUC* Area under curve

**Table 5:** Other QoL measures rather than SF / RAND, as predictor of all-cause mortality

Author (Year)	Comparison	Effect estimate (95% CI)
**Core CDC Healthy Days Measures (HRQOL-4) (General Health) categorised**		
Brown et al. 2015^a^ [38]	HR, Excellent vs. Poor	0.24 (0.21–0.27)
Dominick et al. 2002^a^ [44]	RR, Excellent vs. Poor	0.24 (0.17–0.33)
**WHO QOL – BREF (Overall)**		
Kao et al. 2005 [54]	RR, 1-point change	0.99 (0.77–1.26)
Murray et al. 2011 [63]	HR, 1-tertile increase	0.84 (0.67–1.05)
**WHO QOL (Categorised)**		
Gomez-Olive et al. 2014^a^ [25]	HR, Highest vs. Lowest	0.61
Razzaque et al. 2014^a^ [69]	RR, Good vs. Bad	0.26 (0.16–0.41) men 0.30 (0.10–0.86) women
**Psychological General Well-being (PGWB) (Global Score) continuous**		
Nilsson et al. 2011^a^ [66]	RR, 1-unit change	0.984 (0.969–0.998) men 0.994 (0.978–1.010) women
**Lancashire Quality-of-life Profile-Residential (LQOLP-R) incorporated the Spitzer Uniscale**		
Sutcliffe et al. 2007 [72]	HR, increased score	0.9805 (0.9704–0.9907)
**Chinese 35-item Quality of Life (QOL-35) categorised**		
Xie et al. 2014^a^ [77]	HR, Upper 50% vs. Lower 50%	0.69 (0.49–1.00)
**The Health Utilities Index Mark 3 Version (HUI3) continuous**		
Feeny et al. 2012 [48]	HR, 1-level increase	Hearing: 0.18 (0.06–0.57) Ambulation: 0.10 (0.04–0.23) Pain: 0.53 (0.29–0.96)
Kaplan et al. 2007 [55]	HR, 1-unit increase	Overall: 0.61 (0.42–0.89)
**The EuroQoL-5 Dimension (EQ-5D) continuous**		
Cavrini et al. 2012 [39]	HR, 1-unit increase	0.42 (0.35–0.50)
**The EuroQoL-5 Dimension EQ-5D categorised**		
Jia et al. 2018^a^ [53]	HR, 5th Quintile vs. 1st Quintile	0.45 (0.43–0.49)
**Short Form Six Dimension Utility Index (SF-6D) continuous**		
Myint et al. 2010^a^ [26]	HR, 1SD 0.12-point increase	0.74 (0.69–0.79)
**Short Form Six Dimension Health Utility Measure (SF-6D) categorised**		
Jia et al. 2018^a^ [53]	HR, 5th Quintile vs. 1st Quintile	0.77 (0.71–0.80)
**Goteborg Quality of Life Assessment**		
Tibblin et al. 1993 [73]	Only Health variable was significantly related to mortality (No data available)	

^a^where studies report reverse association or risk estimate per more than 1-unit increase, the risk estimates were standardised per 1-unit increase or 1-SD increase or high vs. low for the purpose of consistency across the table

### Meta-analyses

**Fig. 2:**
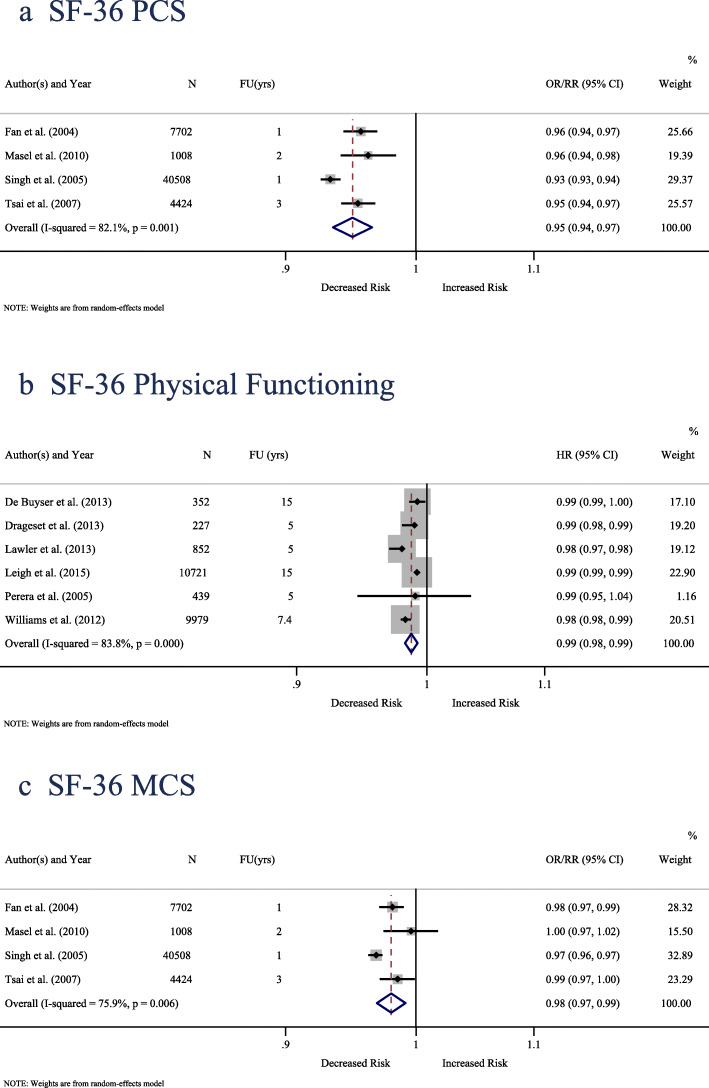
Forest plot of all-cause mortality risk per one unit increase in **a** SF-36 PCS, **b** SF-36 Physical-Functioning, **c** SF-36 MCS. CI = confidence interval; FU (yrs) = follow-up in years; N = sample size; OR = odds ratio; RR = relative risk; HR = hazard ratio

Four studies including 53,642 participants [23, 24, 60, 70] measured QoL using the SF-36 and examined the association between the PCS and all-cause mortality and provided estimates from logistic regression analysis (OR or RR). With an average 1.8-year follow-up, one unit increase in the SF-36 PCS was associated with a 5% decrease in all-cause mortality (pooled OR/RR = 0.950; 95% CI: 0.935 to 0.965; *P*-value < 0.001). There was substantial heterogeneity between studies (I^2^ = 82.1%; *P*-value = 0.001) (Fig. 2-a).

Six studies including 22,570 participants [42, 46, 57, 59, 68, 76] measured QoL using the SF-36 and investigated the association between the physical functioning and all-cause mortality using time-to-event survival analysis. With an average 8.7-year follow-up, one unit increase in the SF-36 PF was associated with a 1.3% decrease in time to death (pooled HR = 0.987; 95%CI: 0.982 to 0.992; *P*-value < 0.001). There was substantial heterogeneity between studies (I^2^ = 83.8%; *P*-value < 0.001) (Fig. 2-b).

Four studies including 53,642 participants [23, 24, 60, 70] measured QoL using the SF-36 and examined the association between the MCS and all-cause mortality reported estimates on logistic regression analysis (OR or RR). With an average 1.8-year follow-up, one unit increase in the SF-36 MCS was associated with a 2% decrease in all-cause mortality (pooled OR/RR = 0.980; 95% CI: 0.969 to 0.992; *P*-value = 0.001). There was substantial heterogeneity between studies (I^2^ = 75.9%; *P*-value = 0.01) (Fig. 2-c).

Given the heterogeneity identified in the three meta-analyses described above, the influence of individual studies on the pooled risk estimate was assessed. The removal of no single study affected the association (Supplementary Table S8 – S10, Additional File 1).

**Fig. 3:**
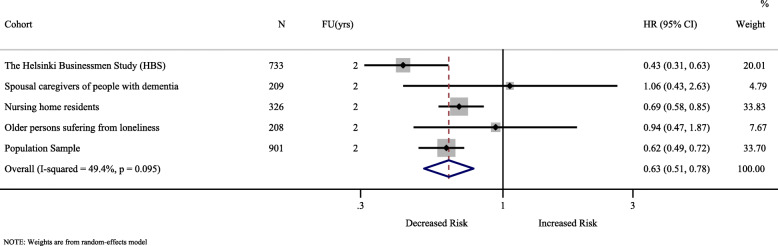
Forest plot of all-cause mortality risk per one-SD (0.14) increase in 15D index. CI = confidence interval; FU (yrs) = follow-up in years; HR = hazard ratio; N = sample size

Five Finnish individual cohorts of the Liira et al. study including 2377 [29] measured QoL using the 15D index and explored its association with all-cause mortality using time-to-event survival analysis. With an average 2-year follow-up, one SD (0.14) increase in the 15D index was associated with a 36.7% decrease in all-cause mortality (pooled HR = 0.633; 95%CI: 0.514 to 0.780; *P*-value < 0.001). There was moderate heterogeneity between studies (I^2^ = 49.4%; *P*-value = 0.10) (Fig. 3).

Visual inspection of the funnel plots which were used to assess for publication bias were presented in the Supplementary Figures S1-S4, Additional File 1. For three of the four meta-analyses, there was no strong evidence of publication bias, however for the meta-analysis of MCS, this test was statistically significant (*P* = 0.04).

## Discussion

This systematic review is the first to investigate the association between QoL and mortality in community-dwelling individuals with or without health conditions rather than patients in a hospital or people living in assisted living. It summarizes the findings from 47 studies including approximately 1,200,000 individuals aged predominantly 65 years and older (age range 18–101 years), with 46 studies (98%) conducted in high-income or upper-middle-income countries. Overall thirteen different instruments were used to assess the association between QoL or more specifically HRQoL and mortality risk after 9 months to 18 years of follow-up, with the SF-36 or its derivatives (RAND-36, SF-20, SF-6D) most commonly used. Overall, 43 (91.5%) studies of the 47 included studies reported for at least one of the domains examined, that better QoL was associated lower mortality risk, which was also supported by the results of four meta-analyses (11 studies, *n* = 78,589) of PCS, physical function and MCS domains of the SF-36, and 15D HRQoL.

Our findings are in line with a previous study that used pooled analysis [29] of eight heterogenous Finnish cohorts using the 15D HRQoL measure and included a wide range of both community-dwelling participants with or without morbidity, such as cardiovascular disease, dementia, and hospitalized patients with delirium. They also found that the 15D HRQoL measure was associated with two-year survival, with a slightly higher hazard ratio than that found in our study (HR per 1-SD = 0.44, 95% CI 0.40 to 0.48) [29]. These differences may relate to their inclusion of patient groups in generally poorer health, while our systematic review focused on the community dwelling population. Moreover, our findings in the general non-patient population are also comparable with studies investigating people with specific diseases such as cancer and chronic kidney disease, which reported QoL to be a predictor of mortality risk [19–21].

The findings of the present study are also consistent with those of recent population-based systematic review which investigated on the association between QoL and multimorbidity [78]. In their recent study, Makovski et al. (2019) systematically reviewed the evidence on the relationship between QoL and multimorbidity. They observed a stronger relationship between the PCS of QoL and multimorbidity (overall decline in QoL per additional disease = − 4.37, 95%CI − 7.13% to − 1.61% for WHOQoL-BREF physical domain and − 1.57, 95%CI − 2.70% to − 0.44% for WHOQoL-BREF mental domain) [78]. These findings also align with the results of the present study, where the meta-analysis indicated a stronger effect size for PCS compared to MCS using the SF-36 tool (pooled OR/RR = 0.950; 95% CI: 0.935 to 0.965 for PCS; and pooled OR/RR = 0.980; 95%CI: 0.969 to 0.992 for MCS). Since physical health is generally recognised as a strong risk factor for comorbidity, hospitalisations and mortality [79–82], our findings add further support to the predictive capacity of physical HRQoL for mortality risk. Like other objective health measures such as body mass index, glycaemia, and blood pressure, these findings highlight the utility of assessing physical HRQoL in general clinical practice to help identify individuals at greatest risk of death [83].

Given the evidence regarding the longitudinal relationship between QoL and mortality risk, the utility of a QoL tool in general care may improve patient’ health which in turn would decrease mortality. Furthermore, mental health issues such as depression or anxiety could also be identified through QoL measures and this would enable initiation of early interventions for mental health which in turn could improve long term QoL of individuals. Hence, the finding of this review can help to increase the efficacy of disease prevention strategies in older people through identifying individuals at higher risk for adverse health outcomes in general practice / primary health settings. Thus, the mortality risk prediction by QoL might not be very relevant to younger healthy populations although QoL generic measures were designed to be used across a wide range of populations [84]. There is a need for further studies however, in particular to better understand the influence of gender on these associations, and whether differences could be observed for males and females. Understanding these specific relationships could help identify which particular groups are most at risk and enable specific targeting of interventions to these individuals.

### Strengths of the review

Strengths of this systematic review are that it was performed in a rigorous manner, adhering to strict systematic review guidelines. The protocol was registered with the International prospective register of systematic reviews (PROSPERO), and the review was undertaken in accordance with the preferred reporting items for systematic reviews and meta-analyses (PRISMA) statement. A reproducible and rigorous search strategy using three electronic databases was used, which helped ensure that all relevant articles were included. The literature screening was independently performed by two reviewers, who were also involved in the process of data extraction and methodological quality assessment of the included studies in accordance with NOS. Based on the NOS, all studies received greater than or equal to five out of nine stars, which indicates that there was generally a low risk of bias. Similarly, most studies provided risk estimates that controlled for important factors including current health and socio-economic status. Since our review criteria were not limited to articles with the commonly used QoL (or HRQoL) tools such as the SF-36, this has increased the generalisability of the findings. Therefore, this review has a broad and comprehensive perspective, with results that are rigorous and can be reproduced.

### Limitations of the review

Among included articles, large heterogeneity was observed in terms of country-of-origin, participant characteristics, and evaluation of QoL. The majority of the included articles were conducted in English speaking counties, and restriction to English language articles as part of our inclusion criteria, may impact the generalisability of these findings. Since the different QoL standard tools examine different aspects [33, 85] and are not directly comparable, this made comparison of included studies in data synthesis difficult. There were also some differences in the way the data analysis was performed and the results were presented, reporting OR versus HR for example. In addition, some articles reported the risk estimates by comparing categorical QoL groups while others provided the risk estimates per 1 or more units change in the continuous scale. Hence, the different nature of each QoL scale and inconsistency in risk comparison precluded us from including some articles in the meta-analyses. As such, only 11 studies were included across the four meta-analyses of this systematic review, and the meta-analyses still showed substantial heterogeneity. Therefore, caution should be taken with the interpretation of the overall effect estimates. Moreover, since the numbers of studies included in each meta-analysis were fewer than 10 studies, the results of funnel plots or Egger’s test should also be interpreted with caution. Of particular interest here, it has commonly been reported that gender differences exist in QoL and women of all age groups have lower QoL than their male counterparts [86–90]. However, in this review, it was not possible to perform statistical pooling by gender and age groups due to the different reporting strategies of the reviewed studies. Finally, it is important to consider that although studies of mortality are not directly affected by reverse causation, individuals with severely declining health prior to death, would likely report a decreased HRQoL. An ideal study design would involve excluding individuals who died in the first year of the study, or at least, to run sensitivity analysis to ensure these early deaths were not driving the results. Most of the studies included in this review, did not undertake such analyses. Furthermore, around 10% of the included studies have very short follow-up periods of less than 2 years.

## Conclusion

This is the first systematic review and meta-analysis that has determined whether QoL is associated with mortality in the general non-patient population. In summary, the findings provide evidence that better QoL or HRQoL measured by different tools were associated with lower mortality risk in the general population. Therefore, our findings could be applied more generally to QoL or HRQoL assessed using different instruments. Our unique and first review indicates that QoL measures can be considered as potential screening tools beyond the existing traditional clinical assessment of mortality risk. Additionally, our result also encourages clinicians to incorporate QoL measure into routine data collection of health system which in turn could enable initiation of early primary health care for people at high risk of premature death. Furthermore, this study also adds further support to the predictive capacity of physical HRQoL for mortality risk. Additional research is needed to determine whether these associations differ across gender, and other populations in low- and lower-middle-income countries, who have suffered of a double burden of infectious and chronic diseases, with having difficulties for accessing quality health services. Ultimately these findings suggest the utility of QoL measures to help identify populations at greatest risk of mortality and who might benefit most from routine screening in general practice and possible interventions.

## Supplementary information

**Additional file 1: Figure S1.** Funnel plot of all-cause mortality risk per one unit increase in SF-36 PCS. **Figure S2.** Funnel plot of all-cause mortality risk per one unit increase in SF-36 Physical-Functioning. **Figure S3**. Funnel plot of all-cause mortality risk per one unit increase in SF-36 MCS. **Figure S4.** Funnel plot of all-cause mortality risk per one-SD (0.14) increase in 15D index. **Table S1.** Search Strategy using Ovid MEDLINE 1946 to June 212,019. **Table S2.** Search Strategy using Embase Classic 1947 to June 212,019. **Table S3.** Search Strategy using PsycINFO 1806 to June Week 32,019. **Table S4.** Additional Search Strategy up to June Week 32,019. **Table S5.** The list of excluded articles and reasons for exclusion (*n* = 38). **Table S6.** Appraisal Standard of Newcastle/Ottawa Scale. **Table S7.** Quality appraisal of included studies based on the Newcastle–Ottawa Quality Assessment Scale. **Table S8.** One study removed analysis for all-cause mortality risk per one unit increase in SF-36 PCS. **Table S9.** One study removed analysis for all-cause mortality risk per one unit increase in SF-36 Physical-Functioning. **Table S10.** One study removed analysis for all-cause mortality risk per one unit increase in SF-36 MCS.

## Data Availability

All data generated or analysed during this study are included in this published article (and its supplementary information files).

## Abbreviations

15D: 15-dimentional
CI: Confidence intervals
EQ-5D: Euroqol-5 dimension
HR: Hazard ratio
HRQoL: Health-related quality of life
HUI3: Health utilities index 3
MCS: Mental component score
NOS: NEWCASTLE-Ottawa quality assessment scale
OR: Odds ratio
PCS: Physical component score
PRISMA: Preferred reporting items for systematic reviews and meta-analyses
PROMs: Patient reported outcome measures
PROSPERO: International prospective register of systematic reviews
QoL: Quality of life
RR: Relative risk
SD: Standard deviation
SF-12: 12-items short form
SF-20: 20-item short form
SF-36: 36-item short form
SF-6D: Six-dimension utility index
