# Supplementary material for: Quality of life and mortality in the general population: a systematic review and meta-analysis
Source: BMC Public Health. 2020 Nov 6;20:1596. doi: 10.1186/s12889-020-09639-9 (PMC7646076; doi:10.1186/s12889-020-09639-9)
Supplement: Supplementary file 1 — Additional file 1: Figure S1. Funnel plot of all-cause mortality risk per one unit increase in SF-36 PCS. Figure S2. Funnel plot of all-cause mortality risk per one unit increase in SF-36 Physical-Functioning. Figure S3. Funnel plot of all-cause mortality risk per one unit increase in SF-36 MCS. Figure S4. Funnel plot of all-cause mortality risk per one-SD (0.14) increase in 15D index. Table S1. Search Strategy using Ovid MEDLINE 1946 to June 212,019. Table S2. Search Strategy using Embase Classic 1947 to June 212,019. Table S3. Search Strategy using PsycINFO 1806 to June Week 32,019. Table S4. Additional Search Strategy up to June Week 32,019. Table S5. The list of excluded articles and reasons for exclusion (n = 38). Table S6. Appraisal Standard of Newcastle/Ottawa Scale. Table S7. Quality appraisal of included studies based on the Newcastle–Ottawa Quality Assessment Scale. Table S8. One study removed analysis for all-cause mortality risk per one unit increase in SF-36 PCS. Table S9. One study removed analysis for all-cause mortality risk per one unit increase in SF-36 Physical-Functioning. Table S10. One study removed analysis for all-cause mortality risk per one unit increase in SF-36 MCS. [file 12889_2020_9639_MOESM1_ESM.docx]

**Additional File 1: Supplementary Material**

Figure S1 Funnel plot of all-cause mortality risk per one unit increase in SF-36 PCS.

OR = odds ratio; RR = relative risk

Figure S2 Funnel plot of all-cause mortality risk per one unit increase in SF-36 Physical-Functioning

HR = hazard ratio

Figure S3 Funnel plot of all-cause mortality risk per one unit increase in SF-36 MCS.

OR = odds ratio; RR = relative risk

Figure S4 Funnel plot of all-cause mortality risk per one-SD (0.14) increase in 15D index

HR = hazard ratio

Table S1 Search Strategy using Ovid MEDLINE 1946 to June 21 2019

| **Search Terms** |
| --- |
| mortality/ or "cause of death"/ or fatal outcome/ or hospital mortality/ or perinatal mortality/ or maternal mortality/ or mortality, premature/ or survival rate/ or death/ or brain death/ or death, sudden/ or death, sudden, cardiac/ or karoshi death/ or parental death/ or maternal death/ or perinatal death/ |
| AND |
| life quality or quality of life or hrqol or 36-Item Short-Form or SF-36 or Veterans Short Form 36 or SF-36 V or 12-Item Short-Form or SF-12 or RAND-36 or 4-item Healthy Days Core Module or CDC HRQOL-4 |
| AND |
| cohort studies/ or follow-up studies/ or longitudinal studies/ or prospective studies/ or retrospective studies/ |

Table S2 Search Strategy using Embase Classic 1947 to June 21 2019

| **Search Terms** |
| --- |
| mortality/ or all cause mortality/ or cancer mortality/ or cardiovascular mortality/ or hospital mortality/ or maternal mortality/ or mortality rate/ or perinatal mortality/ or premature mortality/ or prenatal mortality/ or standardized mortality ratio/ or surgical mortality/ or death/ or accidental death/ or brain death/ or "cause of death"/ or dying/ or fatality/ or heart death/ or lethality/ or parental death/ or perinatal death/ or sudden death/ |
| AND |
| life quality or quality of life or hrqol or 36-Item Short-Form or SF-36 or Veterans Short Form 36 or SF-36 V or 12-Item Short-Form or SF-12 or RAND-36 or 4-item Healthy Days Core Module or CDC HRQOL-4 |
| AND |
| clinical study/ or longitudinal study/ or prospective study/ or retrospective study/ or cohort analysis/ or follow up/ |

Table S3 Search Strategy using PsycINFO 1806 to June Week 3 2019

| **Search Terms** |
| --- |
| "death and dying"/ or brain death/ or sudden death/ or mortality risk/ |
| AND |
| life quality or quality of life or hrqol or 36-Item Short-Form or SF-36 or Veterans Short Form 36 or SF-36 V or 12-Item Short-Form Health Survey or SF-12 or RAND-36 or 4-item Healthy Days Core Module or CDC HRQOL-4 |
| AND |
| experimental design/ or cohort analysis/ or follow up studies/ or longitudinal studies/ or retrospective studies/ or prospective studies |

Table S4 Additional Search Strategy up to June Week 3 2019

| **Search Terms** |
| --- |
| **Ovid MEDLINE** |
| mortality/ or "cause of death"/ or fatal outcome/ or hospital mortality/ or perinatal mortality/ or maternal mortality/ or mortality, premature/ or survival rate/ or death/ or brain death/ or death, sudden/ or death, sudden, cardiac/ or karoshi death/ or parental death/ or maternal death/ or perinatal death/ |
| AND |
| EuroQoL-5 Dimension or EQ-5D or short form six-dimension utility index or SF-6D or The Health Utilities Index 3 or HUI3 or Psychological General Well-Being Index or PGWB or 20-Item Short-Form or SF-20 or World Health Organization Quality of Life or WHOQOL or WHOQOL-BREF or 15-dimensional index or 15D or Goteborg Quality of Life Instrument or Goteborg QoL or Lancashire Quality of Life Profile or LQOLP or Chinese 35-Item Quality of Life Instrument or QOL-35 |
| AND |
| cohort studies/ or follow-up studies/ or longitudinal studies/ or prospective studies/ or retrospective studies/ |
| **EMBASE Classic** |
| mortality/ or all cause mortality/ or cancer mortality/ or cardiovascular mortality/ or hospital mortality/ or maternal mortality/ or mortality rate/ or perinatal mortality/ or premature mortality/ or prenatal mortality/ or standardized mortality ratio/ or surgical mortality/ or death/ or accidental death/ or brain death/ or "cause of death"/ or dying/ or fatality/ or heart death/ or lethality/ or parental death/ or perinatal death/ or sudden death/ |
| AND |
| EuroQoL-5 Dimension or EQ-5D or short form six-dimension utility index or SF-6D or The Health Utilities Index 3 or HUI3 or Psychological General Well-Being Index or PGWB or 20-Item Short-Form or SF-20 or World Health Organization Quality of Life or WHOQOL or WHOQOL-BREF or 15-dimensional index or 15D or Goteborg Quality of Life Instrument or Goteborg QoL or Lancashire Quality of Life Profile or LQOLP or Chinese 35-Item Quality of Life Instrument or QOL-35 |
| AND |
| clinical study/ or longitudinal study/ or prospective study/ or retrospective study/ or cohort analysis/ or follow up/ |
| **PsycINFO** |
| "death and dying"/ or brain death/ or sudden death/ or mortality risk/ |
| AND |
| EuroQoL-5 Dimension or EQ-5D or short form six-dimension utility index or SF-6D or The Health Utilities Index 3 or HUI3 or Psychological General Well-Being Index or PGWB or 20-Item Short-Form or SF-20 or World Health Organization Quality of Life or WHOQOL or WHOQOL-BREF or 15-dimensional index or 15D or Goteborg Quality of Life Instrument or Goteborg QoL or Lancashire Quality of Life Profile or LQOLP or Chinese 35-Item Quality of Life Instrument or QOL-35 |
| AND |
| experimental design/ or cohort analysis/ or follow up studies/ or longitudinal studies/ or retrospective studies/ or prospective studies |

Table S5 The list of excluded articles and reasons for exclusion (n = 38)

| **List of Excluded Articles** | **Reasons for exclusion** |
| --- | --- |
| Basu *et al*. (2017) [1] | No QoL measurement |
| Bobak *et al.* (2004) [2] | Community Comparison and QoL was not studied as predictor of Mortality |
| Bowe *et al.* (2006) [3] | QoL was not studied as predictor of Mortality |
| Boyle *et al.* (2009) [4] | No QoL measurement |
| Byles *et al.* (2006) [5] | QoL was not studied as predictor of Mortality |
| Diehr *et al.* (2003) [6] | Focus was not on Mortality prediction by QoL |
| Fernandez-Ruiz *et al.* (2013) [7] | No QoL measurement |
| Fujino *et al.* (2005) [8] | No QoL measurement |
| Furuya *et al.* (2008) [9] | QoL was not studied as predictor of mortality |
| Gold *et al.* (1996) [10] | QoL was not studied as predictor of mortality |
| Gomez-Olive *et al.* (2014) [11] | Do not use QoL to predict mortality and mortality was mortality over time associated with sleep problems |
| Hanly *et al.* (2016) [12] | Majority are patients and QoL was not studied to predict mortality |
| Hartog *et al.* (2016) [13] | Patient sample population |
| Holahan *et al.* (2013) [14] | QoL was not studied as predictor of mortality |
| Huohvanainen *et al.* (2016) [15] | QoL was not studied as predictor of mortality |
| Hwang *et al.* (2004) [16] | Patient sample population |
| Kakizaki *et al.* (2013) [17] | QoL was not studied as predictor of mortality |
| Ladwig *et al.* (2004) [18] | QoL was not studied as predictor of mortality |
| Lee *et al.* (2000) [19] | No QoL measurement |
| Lopez *et al.* (2011) [20] | QoL was not studied as predictor of mortality |
| Mesas *et al.* (2010) [21] | QoL was not studied as predictor of mortality |
| Miller *et al.* (2007) [22] | No QoL measurement |
| Mold *et al.* (2011) [23] | Illness Subgroup analysis and the same study with the included study – Mold *et al.* (2008) |
| Mora *et al.* (2013) [24] | No QoL measurement |
| Mukamal *et al.* (2007) [25] | QoL was not studied as predictor of mortality |
| Myint *et al.* (2011) [26] | QoL was not studied as predictor of mortality and same study with the included studies – Myint *et al.* (2006) (2007) (2010) |
| Pinheiro *et al.* (2019) [27] | Outcome was not Mortality |
| Rahman *et al.* (2019) [28] | Outcome was not Mortality |
| Rakowski *et al.* (1993) [29] | No QoL measurement |
| Rosero-Bixby *et al.* (2009) [30] | QoL was not studied as predictor of mortality |
| Strandberg *et al.* (2017) [31] | QoL was not studied as predictor of mortality |
| Svardsudd *et al.* (1990) [32] | No full-text available |
| Urtamo *et al.* (2018) [33] | QoL was not studied as predictor of mortality |
| Wei *et al.* (2019) [34] | QoL was not studied as predictor of mortality |
| Wolf *et al.* (2010) [35] | QoL was not studied as predictor of mortality |
| Wong *et al.* (2013) [36] | QoL was not studied as predictor of mortality |
| Wu *et al.* (2016) [37] | QoL was not studied as predictor of mortality |
| Zhu *et al.* (2007) [38] | QoL was not studied as predictor of mortality |

Table S6 Appraisal Standard of Newcastle/Ottawa Scale

| **Content** |
| --- |
| **Selection** |
| 1. **Representativeness of the exposed cohort** 2. Truly representative of the average general population with or without health conditions * 3. Somewhat representative of the average general population with or without health conditions * 4. Selected group of users 5. No description of the derivation of the cohort |
| 1. **Selection of the non-exposed group/cohort** 2. Drawn from the same community as the exposed cohort * 3. Drawn from a different source 4. No description of the derivation of the non-exposed group |
| 1. **Ascertainment of exposure** 2. Secure record * 3. Structured interview or questionnaire * 4. Written self-reports 5. No description |
| 1. **Demonstration that outcome of interest was not present at the start of study** 2. Yes * 3. No |
| **Comparability** |
| 1. **Comparability of cohorts on the basis of the design or analysis** 2. Study control for most important factors – age, sex, socioeconomic status AND some measure of comorbidity* 3. Study control for any additional factors – clinical measure (could be BMI) or lifestyle factors (could be smoking and / or alcohol and / or physical activity) * |
| **Outcome** |
| 1. **Assessment of outcome** 2. Independent blind assessment * 3. Record linkage * 4. Self-report 5. No description |
| 1. **Was follow-up long enough for outcomes to occur** 2. Yes (More than and equal to one-year follow-up) * 3. No |
| 1. **Adequacy of follow-up of cohorts** 2. Complete follow up – all subjects accounted for * 3. Subjects lost to follow-up unlikely to introduce bias – small number lost - > 80% follow up, or description provided for those lost * 4. Follow-up < 80% and no description of those lost 5. No statement |

*1 point; a maximum score of 9; 9 stars indicates high methodological quality. The range of NOS scoring is from 0 to 9 stars, with higher scores indicating less susceptibility to bias.

Table S7 Quality appraisal of included studies based on the Newcastle–Ottawa Quality Assessment Scale

| **Authors** | **Year** | **Selection** | | | | **Comparability** | | **Outcome** | | | **Overall**  **(9 scores)** |
| --- | --- | --- | --- | --- | --- | --- | --- | --- | --- | --- | --- |
|  |  | **1** | **2** | **3** | **4** | **1** | | **1** | **2** | **3** |  |
| Bjorkman *et al.* | 2019 | (a)* | (a)* | (c) | (a)* |  | (b)* | (b)* | (a)* | (a)* | 7 |
| Brown *et al.* | 2015 | (b)* | (a)* | (c) | (a)* | (a)* | (b)* | (b)* | (a)* | (a)* | 8 |
| Cavrini *et al.* | 2012 | (a)* | (a)* | (c) | (a)* | (a)* | (b)* | (b)* | (a)* | (a)* | 8 |
| Chwastiak *et al.* | 2010 | (c) | (a)* | (c) | (a)* | (a)* | (b)* | (b)* | (a)* | (a)* | 7 |
| De Buyser *et al.* | 2016 | (a)* | (a)* | (b)* | (a)* |  | (b)* | (b)* | (a)* | (a)* | 8 |
| De Buyser *et al.* | 2013 | (a)* | (a)* | (b)* | (a)* |  | (b)* | (b)* | (a)* | (a)* | 8 |
| DeSalvo *et al.* | 2005 | (c) | (a)* | (c) | (a)* |  |  | (b)* | (a)* | (a)* | 5 |
| Dominick *et al.* | 2002 | (c) | (a)* | (c) | (a)* | (a)* |  | (b)* | (a)* | (a)* | 6 |
| Dorr *et al.* | 2006 | (b)* | (a)* | (c) | (b) |  | (b)* | (b)* | (a)* | (a)* | 6 |
| Drageset *et al.* | 2013 | (b)* | (a)* | (c) | (b) | (a)* |  | (b)* | (a)* | (a)* | 6 |
| Fan *et al.* | 2004 | (c) | (a)* | (c) | (a)* |  | (b)* | (b)* | (a)* | (a)* | 6 |
| Fan *et al.* | 2006 | (c) | (a)* | (c) | (a)* |  |  | (b)* | (a)* | (a)* | 5 |
| Feeny *et al.* | 2012 | (a)* | (a)* | (b)* | (a)* | (a)* | (b)* | (b)* | (a)* | (a)* | 9 |
| Forsyth *et al.* | 2018 | (c) | (a)* | (b)* | (a)* |  |  | (b)* | (a)* | (a)* | 6 |
| Franks *et al.* | 2003 | (a)* | (a)* | (c) | (a)* |  |  | (b)* | (a)* | (a)* | 6 |
| Gomez-Olive *et al.* | 2014 | (a)* | (a)* | (b)* | (a)* | (a)* |  | (d) | (a)* | (a)* | 7 |
| Han *et al.* | 2009 | (a)* | (a)* | (b)* | (a)* |  | (b)* | (b)* | (a)* | (a)* | 8 |
| Haring *et al.* | 2011 | (a)* | (a)* | (b)* | (a)* | (a)* | (b)* | (d) | (a)* | (a)* | 8 |
| Higueras-Fresnillo *et al.* | 2018 | (a)* | (a)* | (b)* | (a)* | (a)* | (b)* | (b)* | (a)* | (a)* | 9 |
| Jia *et al.* | 2018 | (a)* | (a)* | (c) | (b) | (a)* | (b)* | (b)* | (a)* | (a)* | 7 |
| Kao *et al.* | 2005 | (c) | (a)* | (b)* | (a)* |  | (b)* | (b)* | (a)* | (a)* | 7 |
| Kaplan *et al.* | 2007 | (a)* | (a)* | (b)* | (a)* | (a)* | (b)* | (b)* | (a)* | (a)* | 9 |
| Kroenke *et al.* | 2008 | (c) | (a)* | (c) | (a)* | (a)* | (b)* | (b)* | (a)* | (a)* | 7 |
| Lawler *et al.* | 2013 | (a)* | (a)* | (c) | (a)* | (a)* | (b)* | (b)* | (a)* | (a)* | 8 |
| Lee *et al.* | 2012 | (a)* | (a)* | (b)* | (a)* |  | (b)* | (b)* | (a)* | (a)* | 8 |
| Leigh *et al.* | 2015 | (a)* | (a)* | (c) | (a)* | (a)* | (b)* | (b)* | (a)* | (a)* | 8 |
| Liira *et al.* | 2018 | (b)* | (a)* | (b)* | (a)* |  |  | (b)* | (a)* | (a)* | 7 |
| Masel *et al.* | 2010 | (a)* | (a)* | (b)* | (a)* | (a)* | (b)* | (b)* | (a)* | (a)* | 9 |
| Mold *et al.* | 2008 | (c) | (a)* | (c) | (a)* | (a)* | (b)* | (b)* | (a)* | (a)* | 7 |
| Munoz *et al.* | 2011 | (a)* | (a)* | (b)* | (a)* | (a)* | (b)* | (b)* | (a)* | (a)* | 9 |
| Murray *et al.* | 2011 | (a)* | (a)* | (c) | (a)* | (a)* | (b)* | (b)* | (a)* | (a)* | 8 |
| Myint *et al.* | 2006 | (a)* | (a)* | (c) | (a)* | (a)* | (b)* | (b)* | (a)* | (a)* | 8 |
| Myint *et al.* | 2007 | (a)* | (a)* | (c) | (a)* | (a)* | (b)* | (b)* | (a)* | (a)* | 8 |
| Myint *et al.* | 2010 | (a)* | (a)* | (c) | (a)* | (a)* | (b)* | (b)* | (a)* | (a)* | 8 |
| Nilsson *et al.* | 2011 | (a)* | (a)* | (c) | (a)* |  | (b)* | (b)* | (a)* | (a)* | 7 |
| Otero-Rodriguez *et al.* | 2010 | (a)* | (a)* | (b)* | (a)* | (a)* | (b)* | (b)* | (a)* | (a)* | 9 |
| Perera *et al.* | 2005 | (b)* | (a)* | (b)* | (a)* |  | (b)* | (b)* | (a)* | (a)* | 8 |
| Razzaque *et al.* | 2-14 | (a)* | (a)* | (b)* | (a)* |  |  | (b)* | (a)* | (a)* | 7 |
| Singh *et al.* | 2005 | (b)* | (a)* | (c) | (b) | (a)* | (b)* | (b)* | (a)* | (a)* | 7 |
| St.John *et al.* | 2018 | (c) | (a)* | (c) | (a)* |  |  | (b)* | (a)* | (a)* | 5 |
| Sutcliffe *et al.* | 2007 | (c) | (a)* | (b)* | (a)* |  |  | (b)* | (b) | (a)* | 5 |
| Tibblin *et al.* | 1993 | (a)* | (a)* | (b)* | (a)* |  | (b)* | (b)* | (b)* | (a)* | 8 |
| Tice *et al.* | 2006 | (a)* | (a)* | (c) | (a)* |  | (b)* | (b)* | (a)* | (a)* | 7 |
| Tsai *et al.* | 2007 | (a)* | (a)* | (b)* | (a)* |  | (b)* | (b)* | (a)* | (a)* | 8 |
| Ul-Haq *et al.* | 2014 | (a)* | (a)* | (b)* | (a)* | (a)* | (b)* | (b)* | (a)* | (a)* | 9 |
| Williams *et al.* | 2012 | (a)* | (a)* | (b)* | (a)* |  | (b)* | (b)* | (a)* | (a)* | 8 |
| Xie *et al.* | 2014 | (a)* | (a)* | (c) | (a)* | (a)* | (b)* | (b)* | (a)* | (a)* | 8 |

a, b, c, and d are the categories of each numbered item within each domain (Selection, Comparability, and Outcome) of NOS, in order of highest to lowest quality. A Star was given based on the appraisal standard of NOS. Blank cells in comparability domain indicate that the study was not controlled for (a) important covariates – age, sex, socioeconomic status or proxy and some measure of co-morbidity, and / or (b) additional health conditions including clinical measures, BMI, or lifestyle factors.

Table S8 One study removed analysis for all-cause mortality risk per one unit increase in SF-36 PCS

| Author (Year) | Pooled Estimates* | 95% Confidence Intervals (CI) * |
| --- | --- | --- |
| Fan et al. (2004) | 0.948 | (0.930, 0.966) |
| Masel et al. (2010) | 0.947 | (0.931, 0.964) |
| Singh et al. (2005) | 0.956 | (0.948, 0.965 |
| Tsai et al. (2007) | 0.949 | (0.929, 0.968) |

*Each pooled estimates and 95%CIs represent the results of meta-analysis of all studies except the omitted study named in that row

Table S9 One study removed analysis for all-cause mortality risk per one unit increase in SF-36 Physical-Functioning

| Author (Year) | Pooled Estimates* | 95% Confidence Intervals (CI) * |
| --- | --- | --- |
| De Buyser et al. (2013) | 0.986 | (0.981, 0.992) |
| Drageset et al. (2013) | 0.987 | (0.981, 0.993) |
| Lawler et al. (2013) | 0.989 | (0.985, 0.994) |
| Leigh et al. (2015) | 0.986 | (0.981, 0.991) |
| Perera et al. (2005) | 0.987 | (0.982, 0.993) |
| Williams et al. (2012) | 0.989 | (0.983, 0.994) |

*Each pooled estimates and 95%CIs represent the results of meta-analysis of all studies except the omitted study named in that row

Table S10 One study removed analysis for all-cause mortality risk per one unit increase in SF-36 MCS

| Author (Year) | Pooled Estimates* | 95% Confidence Intervals (CI) * |
| --- | --- | --- |
| Fan et al. (2004) | 0.981 | (0.964, 0.997) |
| Masel et al. (2010) | 0.977 | (0.966, 0.988) |
| Singh et al. (2005) | 0.984 | (0.977, 0.992) |
| Tsai et al. (2007) | 0.979 | (0.965, 0.992) |

*Each pooled estimates and 95%CIs represent the results of meta-analysis of all studies except the omitted study named in that row

**References for Supplementary Material**

1. Basu N, Yang X, Luben RN, Whibley D, Macfarlane GJ, Wareham NJ, et al. Lowvitality predicts mortality in the epic-norfolk population-based study. Age and Ageing. 2017;46(Supplement 1).

2. Bobak M, Kristenson M, Pikhart H, Marmot M. Life span and disability: a cross sectional comparison of Russian and Swedish community based data. BMJ (Clinical research ed). 2004;329(7469):767.

3. Bowe S, Young AF, Sibbritt D, Furuya H. Transforming the SF-36 to account for death in longitudinal studies with three-year follow-up. Medical care. 2006;44(10):956-9.

4. Boyle PA, Barnes LL, Buchman AS, Bennett DA. Purpose in life is associated with mortality among community-dwelling older persons. Psychosomatic Medicine. 2009;71(5):574-9.

5. Byles J, Young A, Furuya H, Parkinson L. A drink to healthy aging: The association between older women's use of alcohol and their health-related quality of life. Journal of the American Geriatrics Society. 2006;54(9):1341-7.

6. Diehr P, Patrick DL, McDonell MB, Fihn SD. Accounting for deaths in longitudinal studies using the SF-36: the performance of the Physical Component Scale of the Short Form 36-item health survey and the PCTD. Medical care. 2003;41(9):1065-73.

7. Fernandez-Ruiz M, Guerra-Vales JM, Trincado R, Fernandez R, Medrano MJ, Villarejo A, et al. The ability of self-rated health to predict mortality among community-dwelling elderly individuals differs according to the specific cause of death: Data from the NEDICES cohort. Gerontology. 2013;59(4):368-77.

8. Fujino Y, Mizoue T, Tokui N, Yoshimura T. Prospective Cohort Study of Stress, Life Satisfaction, Self-Rated Health, Insomnia, and Suicide Death in Japan. Suicide and Life-Threatening Behavior. 2005;35(2):227-37.

9. Furuya H, Young AF, Powers JR, Byles JE. Alcohol consumption and physical health-related quality of life in older women using the transformation of SF-36 to account for death. Nihon Arukoru Yakubutsu Igakkai zasshi = Japanese journal of alcohol studies & drug dependence. 2008;43(2):97-109.

10. Gold M, Franks P, Erickson P. Assessing the health of the nation. The predictive validity of a preference-based measure and self-rated health. Medical care. 1996;34(2):163-77.

11. Gomez-Olive FX, Thorogood M, Bocquier P, Mee P, Kahn K, Berkman L, et al. Social conditions and disability related to the mortality of older people in rural south africa. International Journal of Epidemiology. 2014;43(5):1531-41.

12. Hanly JG, O'Keeffe AG, Su L, Urowitz MB, Romero-Diaz J, Gordon C, et al. The frequency and outcome of lupus nephritis: results from an international inception cohort study. Rheumatology (Oxford, England). 2016;55(2):252-62.

13. Hartog LC, Landman GWD, Cimzar-Sweelssen M, Knipscheer A, Groenier KH, Kleefstra N, et al. Health-related quality of life, rehabilitation and mortality in a nursing home population. The Netherlands journal of medicine. 2016;74(6):247-56.

14. Holahan CK, Holahan CJ, North RJ, Hayes RB, Powers DA, Ockene JK. Smoking status, physical health-related quality of life, and mortality in middle-aged and older women. Nicotine & Tobacco Research. 2013;15(3):662-9.

15. Huohvanainen E, Strandberg AY, Stenholm S, Pitkala KH, Tilvis RS, Strandberg TE. Association of Self-Rated Health in Midlife With Mortality and Old Age Frailty: A 26-Year Follow-Up of Initially Healthy Men. The journals of gerontology Series A, Biological sciences and medical sciences. 2016;71(7):923-8.

16. Hwang J-S, Wang J-D. Integrating health profile with survival for quality of life assessment. Quality of life research : an international journal of quality of life aspects of treatment, care and rehabilitation. 2004;13(1):1-4.

17. Kakizaki M, Kuriyama S, Nakaya N, Sone T, Nagai M, Sugawara Y, et al. Long sleep duration and cause-specific mortality according to physical function and self-rated health: The Ohsaki Cohort Study. Journal of Sleep Research. 2013;22(2):209-16.

18. Ladwig KH, Marten-Mittag B, Baumert J, Lowel H, Doring A. Case-finding for depressive and exhausted mood in the general population: Reliability and validity of a symptom-driven diagnostic scale. Results from the prospective MONICA/KORA Augsburg study. Annals of Epidemiology. 2004;14(5):332-8.

19. Lee Y. The predictive value of self assessed general, physical, and mental health on functional decline and mortality in older adults. Journal of Epidemiology and Community Health. 2000;54(2):123.

20. Lopez D, McCaul KA, Hankey GJ, Norman PE, Almeida OP, Dobson AJ, et al. Falls, injuries from falls, health related quality of life and mortality in older adults with vision and hearing impairment--is there a gender difference? Maturitas. 2011;69(4):359-64.

21. Mesas AE, Lopez-Garcia E, Leon-Aunoz LM, Guallar-Aastillon P, Rodriguez-Artalejo F. Sleep duration and mortality according to health status in older adults. Journal of the American Geriatrics Society. 2010;58(10):1870-7.

22. Miller TR, Wolinsky FD. Self-rated health trajectories and mortality among older adults. The Journals of Gerontology: Series B: Psychological Sciences and Social Sciences. 2007;62(1):S22-S7.

23. Mold JW, Lawler F, Schauf KJ, Aspy CB. Does patient assessment of the quality of the primary care they receive predict subsequent outcomes? An Oklahoma Physicians Resource/Research Network (OKPRN) study. Journal of the American Board of Family Medicine : JABFM. 2011;24(5):511-23.

24. Mora PA, Orsak G, DiBonaventura MD, Leventhal EA. Why do comparative assessments predict health? The role of self-assessed health in the formation of comparative health judgments. Health Psychology. 2013;32(11):1175-8.

25. Mukamal KJ, Kawachi I, Miller M, Rimm EB. Body mass index and risk of suicide among men. Archives of internal medicine. 2007;167(5):468-75.

26. Myint PK, Smith RD, Luben RN, Surtees PG, Wainwright NWJ, Wareham NJ, et al. Lifestyle behaviours and quality-adjusted life years in middle and older age. Age and ageing. 2011;40(5):589-95.

27. Pinheiro LC, Reshetnyak E, Sterling MR, Richman JS, Kern LM, Safford MM. Using health-related quality of life to predict cardiovascular disease events. Quality of Life Research. 2019;28(6):1465-75.

28. Rahman M, Efird JT, Byles JE. Patterns of aged care use among older Australian women: A prospective cohort study using linked data. Archives of gerontology and geriatrics. 2019;81:39-47.

29. Rakowski W, Fleishman JA, Mor V, Bryant SA. Self-Assessments of Health and Mortality among Older Persons: Do Questions Other than Global Self-Rated Health Predict Mortality? Research on Aging. 1993;15(1):92-116.

30. Rosero-Bixby L, Dow WH. Surprising SES Gradients in mortality, health, and biomarkers in a Latin American population of adults. The journals of gerontology Series B, Psychological sciences and social sciences. 2009;64(1):105-17.

31. Strandberg TE, von Bonsdorff M, Strandberg A, Pitkala K, Raikkonen K. Associations of vacation time with lifestyle, long-term mortality and health-related quality of life in old age: The Helsinki Businessmen Study. European Geriatric Medicine. 2017;8(3):260-4.

32. Svardsudd K, Tibblin G. Is quality of life affecting survival? The study of men born in 1913. Scandinavian journal of primary health care Supplement. 1990;1:55-60.

33. Urtamo A, Kautiainen H, Pitkala KH, Strandberg TE. Association of midlife value priorities with health-related quality of life, frailty and mortality among older men: a 26-year follow-up of the Helsinki Businessmen Study (HBS). Quality of Life Research. 2018;27(5):1269-75.

34. Wei MY, Mukamal KJ. Multimorbidity and mental health-related quality of life and risk of completed suicide. Journal of the American Geriatrics Society. 2019;67(3):511-9.

35. Wolf MS, Feinglass J, Thompson J, Baker DW. In search of 'low health literacy': threshold vs. gradient effect of literacy on health status and mortality. Social science & medicine (1982). 2010;70(9):1335-41.

36. Wong YYE, Almeida OP, McCaul KA, Yeap BB, Hankey GJ, Flicker L. Homocysteine, frailty, and all-cause mortality in older men: The Health in Men Study. The Journals of Gerontology: Series A: Biological Sciences and Medical Sciences. 2013;68(5):590-8.

37. Wu TY, Liaw CK, Chen FC, Kuo KL, Chie WC, Yang RS. Sarcopenia Screened With SARC-F Questionnaire Is Associated With Quality of Life and 4-Year Mortality. Journal of the American Medical Directors Association. 2016;17(12):1129-35.

38. Zhu K, Devine A, Dick IM, Prince RL. Association of back pain frequency with mortality, coronary heart events, mobility, and quality of life in elderly women. Spine. 2007;32(18):2012-8.
